# Comparative oncology in action: vignettes on immunotherapy development

**DOI:** 10.1186/s44356-025-00017-4

**Published:** 2025-02-14

**Authors:** Amy LeBlanc, Christina N. Mazcko, Nicola J. Mason, M. Renee Chambers, David M. Brockington, G. Elizabeth Pluhar, Shruthi Naik

**Affiliations:** 1 Comparative Oncology Program, Center for Cancer Research, National Cancer Institute, National Institutes of Health, 10 Center Drive, Room 1B58, Bethesda, MD 20892 USA; 2 Comparative Immunotherapy Program, Department of Pathobiology, School of Veterinary Medicine, University of Pennsylvania, Pennsylvania, PA USA; 3 Department of Neurological Surgery, University of Alabama at Birmingham, Birmingham, AL USA; 4 Department of Veterinary Clinical Sciences, College of Veterinary Medicine, University of Minnesota, St Paul, MN USA; 5 Department of Molecular Medicine, Mayo Clinic, Rochester, MN USA

**Keywords:** Comparative oncology, Drug development, Clinical trials, Immunotherapy

## Abstract

Immunotherapeutic approaches to cancer treatment have gained significant traction in recent years, due in large part to the success of immune checkpoint inhibitors and T cell-based therapies. Comparative oncology is the study of naturally-occurring cancer in companion (pet) animals, mainly dogs, and is a powerful tool in cancer research and drug development. Given their intact, educated immune systems and natural co-evolution of tumor, microenvironment and stromal components, tumor-bearing pet dogs are an attractive species in which to explore these cellular interactions and test novel therapeutic approaches. Moreover, similarities between the canine and human immune systems support assessment of a wide variety of approaches, including antagonistic or agonistic antibodies directed at specific cellular targets, tumor vaccines, cell-based therapies, and combinations of these with conventional cancer treatments such as chemotherapy and radiotherapy. This manuscript provides specific examples of how canine immunotherapeutic studies informed an approach destined for human use, with an emphasis on study design, correlative immune assay development and application, and definition of biologic effect.

## Introduction

Immunotherapy has become a significant part of cancer treatment in recent years for both human and veterinary patients [1, 2]. With growing enthusiasm for this approach comes the need for informative model systems to develop and refine the next generation of immunotherapeutic strategies for treatment and prevention of cancer. To date, primarily rodent models have been used in this space [3]. In general, tumors are experimentally-induced and the approach leverages a shared immunologic target and species-specific reagents and/or employs genetically engineered mice that possess a ‘humanized’ immune system [4]. Although these preclinical studies can be informative and mechanistically robust, they fail to recapitulate the natural interplay between a spontaneously-arising tumor and its permissive local microenvironment and dysregulated immune system that support tumor growth and progression.

The shortcomings of rodent models can be overcome at least in part by studies conducted in immune-competent pet dogs that spontaneously develop cancer during their natural lifespan. Canine cancers possess clinical, histologic and molecular features that strongly mirror certain human cancers and are frequently treated with the same chemotherapies as human patients, providing opportunities for translational studies that can determine the safety and efficacy profiles of new cancer treatments in a patient population that closely parallels humans. In these comparative oncology veterinary clinical trials, dogs that naturally develop cancer fill an important gap in the available animal models for immunotherapeutic development by acting as a bridge species between rodents and humans [5, 6]. Moreover, veterinary clinical management that is a key component of longitudinal canine trials supports observation and characterization of immune responses and immune-related adverse events [7]. Serial collection of high-value biologic specimens is also an element of comparative oncology clinical trials. These collections often comprise multi-timepoint matched sets of tumor tissue, normal/tumor-adjacent tissue, and peripheral blood components (serum, plasma, peripheral blood mononuclear cells (PBMCs)), which enable correlative studies aimed at mechanistic understanding of the effect of the immunotherapeutic and target modulation in vivo within spontaneously-developing tumors in an immune-competent host. Such correlative studies provide opportunities for unrivaled correlative biomarker assessment with corresponding adverse events and clinical response data, yielding vital information for human trial design and identifying key determinants of positive clinical outcomes.

Canine immunotherapy studies often mirror prior developments for humans (checkpoints, ACT/CAR-Ts, etc.) but provide a proving ground for new approaches and/or combinatorial strategies that are difficult to prioritize for study in human patients, allow testing in new indications, as well as a tractable mechanism for drug repurposing. In recent years, several research groups have reported results from clinical trials evaluating novel and repurposed immunotherapy drug combinations in a variety of canine cancers, including melanoma, osteosarcoma and brain cancers, demonstrating clinical benefits and tumor responses [8–16]. The first approved cancer vaccine in veterinary medicine was developed for dogs with melanoma, validating an approach that was initially developed in rodent models [17]. A preventative cancer vaccine trial is currently underway evauating a novel frameshift peptide-derived vaccine product in over 800 dogs [18]. Further, the landscape of available tools and reagents that are compatible with canine studies, such as canine specific checkpoint inhibitors and assays that support correlative assessment of immune responses, have improved significantly over the last 5–8 years [19–21].

The comparative assessment of immunotherapy carries distinct challenges along with opportunities [22, 23]. These include the unique aspects of the canine immune system, such as differential cellular responses to immunologic stimuli, specific molecular markers that identify immune cell subsets, and a limited but growing availability of reagents, assays, and computational tools to characterize canine tissues [24–26]. These factors should be considered when designing a study to ensure interpretable data and congruency to the approach in humans as much as possible. Here we highlight selected examples of how a comparative oncology approach played a key role in the advancement of immunotherapeutic approaches designed for human use.

## Vignette #1: Recombinant chimeric HER2/neu expressing *Listeria monocytogenes* for appendicular osteosarcoma (OSA)

### Preclinical summary

Epidermal Growth Factor Receptor-2, (HER2/neu; erbB2) is a membrane bound receptor tyrosine kinase that is detected in 30–60% of human and 40% of canine primary osteosarcoma (OSA) samples [27, 28]. Metastatic OSA lesions are more frequently HER2^+^than primary lesions and HER2 expression in OSA has been linked to more aggressive biological behavior and decreased survival [29–31]. Unlike carcinomas where HER2 is predominantly expressed on the cell membrane, HER2 is predominantly identified in the cytoplasm of malignant osteoblasts and its expression occurs in the absence of erbB2 gene amplification [27]. Because of this, strategies to promote HER2-specific T cell responses are more likely to be effective than antibody targeted therapies against HER2 in the treatment of OSA [32].

*Listeria monocytogenes (Lm)*is a potent inducer of innate and adaptive immunity and can be readily engineered as a vector for immunotherapy [33]. *Lm*preferentially infects antigen presenting cells (APCs) and once inside the phagolysosome, it secretes the pore-forming lysin listeriolysin O (LLO), that enables molecular escape into the cytoplasm and access to the MHC class I processing machinery of the APC [34]. Highly attenuated, live *Lm* strains, engineered to express tumor associated antigens (TAA) fused to a truncated LLO, can therefore induce potent anti-tumor CD8^+^ and CD4^+^ T cell responses that break peripheral tolerance and lead to regression of target-antigen^+^tumors [35–37]. ADXS31-164 is a highly attenuated *Lm*strain that expresses a chimeric construct containing HLA-A2 restricted epitopes of human HER2 fused to LLO [37]. In different primary and metastatic rodent tumor models, ADXS31-164 led to HER2-specific T cell responses and regression of established HER2^+^tumors, delayed the development of metastases, and prolonged overall survival [35, 37]. Effects were shown to be HER2-specific and not induced by *Lm* expressing irrelevant TAAs. Importantly, ADXS31-164 broke peripheral tolerance and delayed development of HER2^+^positive breast cancer in rat HER2 transgenic mice that are immunologically tolerant to rat HER2 [37]. In addition to generation of antigen-specific T cell responses, *Lm*-LLO vectors including ADXS31-164 specifically modulate the tumor microenvironment (TME) by reducing the frequency and suppressive capabilities of regulatory T cells and myeloid derived suppressor cells [37, 38]. However, anti-tumor effects of *Lm*-LLO vectors only occur in the presence of an LLO-fused TAA, suggesting that their effect on the TME alone is insufficient for clinical benefit [37]. A small pilot trial in 18 dogs with HER2^+^ appendicular OSA revealed that ADXS31-164 administered following standard of care (SOC) amputation and adjuvant carboplatin, safely induced HER2-specific IFN- responses, delayed metastases, and increased disease free interval (DFI) and overall survivial (OS) when compared to a historical control group of dogs with HER2^+^appendicular OSA [39]. A second study using a lyophilized form of *Lm*expressing the same HER2 construct present in ADXS31-164, in dogs with appendicular OSA reported similar mild, transient adverse events including nausea, lethargy, and fever as in the ADXS31-164 study [12]. However, due to the nature of the study, effects on DFI and OS were not reported. *Lm* homes to anaerobic environments and although the *Lm* vector strain is rapidly cleared from the liver and spleen of immune compromised IFN-γ^−/−^ mice, several dogs treated with the lyophilized version of the *Lm* vector cultured positive for *Lm*after treatment, and were successfully treated with antibiotics [12, 40].

### Comparative oncology trial

Based on favorable outcome results in the original pilot ADXS31-164 study, a multi-institutional, controlled, clinical trial was performed to confirm vaccine safety and anti-metastatic effect and to further investigate correlative biomarkers of clinical response in dogs with OSA [41]. Clinical trial design was the same as the pilot study although HER2 positivity was not required for entry, only three doses of ADXS31-164 were administered and DFI and OS were compared to a contemporaneously collected control group that received SOC alone.

#### Questions

Does ADXS31-164 exhibit anti-metastatic effects and prolong disease free interval when added to a SOC backbone in dogs with appendicular OS when administered in the setting of minimal residual disease? Can immunological biomarkers of clinical response to ADXS31-164 be identified?

#### Study design

**Fig. 1:**
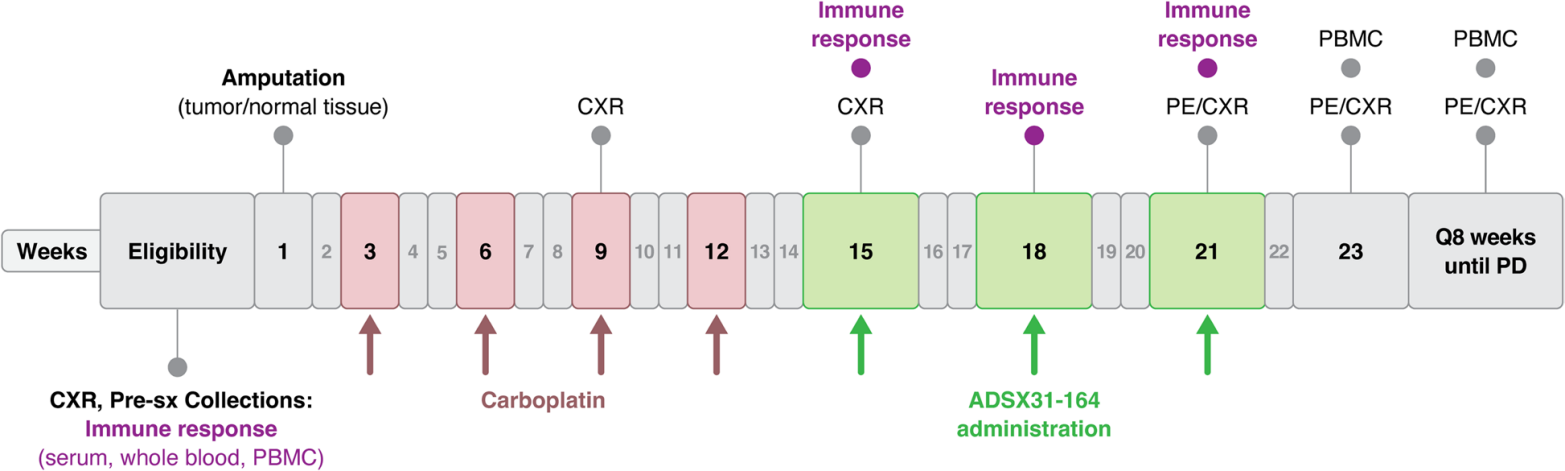
Comparative oncology assessment of ADXS31-164 in dogs with appendicular osteosarcoma. ADXS31-164 therapy was initiated within 7 days after the Week 15 recheck if the canine patient was still free of metastatic disease. Complete blood count (CBC), serum chemistry and urinalysis was performed prior to ADXS31-164 administration and at 3 week intervals until Week 23, with an additional CBC assessed 24 h after each ADXS31-164 dose. Additional correlative immune response data was gathered from serum, whole blood and peripheral blood mononuclear cells (PBMCs) prior to and during ADXS31-164 therapy. PE = physical examination, CXR = 3-view thoracic radiography. PD = progressive disease. Sx = surgery

Study schema are given in Fig. 1. Dogs with appendicular OSA of unknown HER2 status, underwent SOC amputation and received a total of 4 doses of 300 mg/m^2^ carboplatin administered intravenously once every 3 weeks. Dogs confirmed to be free of metastatic disease by thoracic radiographs following carboplatin therapy received 3 doses of 1 × 10^9^ CFUs of ADXS31-164 intravenously once every three weeks. Dogs that completed all three doses of ADXS31-164 and returned for evaluation at week 23 were considered evaluable and were followed for three years with outcomes being compared with the control group that received SOC only.

#### Deliverables

ADXS31-164 was well-tolerated with only transient, low-grade fever, lethargy, nausea, anorexia and diarrhea occurring in the peri-administration period. No clinical listeria infections were reported using ADXS31-164 (not lyophilized) in any of the treated dogs. While the addition of ADXS31-164 to SOC did not significantly prolong DFI or OS, important immunological biomarkers were identified that separated elite and short-term survivors [41]. Elite survivors showed a statistically significant increase in body temperature and serum cytokines including IL-6, TNF-α, IFN-γ and MCP-1 following the first ADXS31-164 vaccination when compared to short-term survivors. The ability of ADXS31-164 to induce fever and innate cytokine production improved in short-term survivors over the course of vaccination. Transcriptomic analysis of PBMCs pre and post ADXS31-164 series revealed a robust cytotoxic response to ADXS31-164 in elite survivors when compared to short-term survivors. These results suggest that elite survivors can be differentiated from short-term survivors by their ability to respond to a potent immunological stimulus, supporting the hypothesis that a patient’s immunological responsiveness or “fitness” at the time of immunotherapy may predict clinical response. These findings provide important correlative biomarkers for investigation in human patients receiving ADXS31-164.

Human use: how was it informed by canine comparative oncology data?

ADXS31-164 (OST31-164) is currently being evaluated in an open-label, multicenter, single-arm phase IIb clinical trial in patients (12–39 years of age) with a recent history of pulmonary recurrent OSA that has been completely resected (NCT0497008). Patients receive OST31-164 every 3 weeks for a total of 48 weeks and are being followed for 3 years. The primary outcome measure is event-free survival at 12 months, and the secondary outcome measure is OS. Incidence of treatment emergent adverse events will also be recorded.

#### Current drug status

OST31-164 received a Rare Pediatric Disease Designation for OSA in November 2021. Enrollment in the phase IIb clinical trial is now complete (39 participants) and patients are in the follow up phase. Full regulatory approval for the lyophilized form of the *Lm*product in dogs with osteosarcoma was not sought by Elanco following assessment of the risks and benefits of the lyophilized product [12].

### Vignette #2: CD200AR-L therapy for malignant glioma

#### Preclinical summary

CD200 is an immune checkpoint protein related to the B7 family of co-stimulatory receptors required for T-cell activation and signaling [42]. CD200 as an immune checkpoint protein was demonstrated in CD200-deficient mice that exhibited auto-immune phenotypes [43]. Importantly, CD200 is expressed on the surface of many types of cancer cells including glioblastoma [44], and can be released in a soluble form (sCD200) when cleaved by metalloproteases. The physical interaction between sCD200 and the inhibitory receptor (CD200R1) on APCs suppresses secretion of pro-inflammatory cytokines and increases production of myeloid-derived suppressor cells (MDSCs) [44] and regulatory T-cells (Tregs) [45, 46] which compromises an anti-tumor immune response.

CD200–CD200R1 interaction is central to maintain the glioblastoma immunosuppressive microenvironment [44]. We developed peptides of CD200 as ligands (CD200AR-L) that mediate their effects by binding to CD200 activation receptors (CD200ARs). We investigated the effects of CD200AR-L alone and in combination with tumor lysate vaccines in a murine glioma (GL261) model. Tumor-bearing mice were given subcutaneous injections of CD200AR-L one day and the injection was repeated 24 h later with and without tumor lysate. We observed statistically significant inhibition in tumor growth and survival benefit only when mice were vaccinated with the combination of CD200AR-L and tumor lysate. Additionally, the anti-tumor response was not seen when a peptide with a scrambled sequence was used [44]. Further studies have demonstrated that CD200R1 and the well-known immune checkpoint protein, PD-1, mediate immune checkpoint signaling through SHIP1. CD200AR-L downregulates expression of CD200R1 and PD-1 and inhibits upregulation of PD-L1 and CTLA4 on antigen-presenting cells (APC) and T-cells.

To prepare for a comparative oncology clinical trial to support a regulatory approval path, we developed canine-specific ligands CD200AR-Ls due to amino acid sequence divergence between the murine and canine CD200ARs and used dosing similar to that in the murine model due to the relative paucity of toxicities associated with immunotherapies. We previously demonstrated safety and efficacy of autologous tumor lysate vaccines in canine glioma patients [47].

### Comparative oncology trial

#### Questions

What are the safety and immunologic features of combining CD200AR-L with autologous tumor lysate vaccination in dogs with spontaneous malignant glioma? Does the addition of CD200AR-L to lysate vaccines provide a significant survival benefit?

#### Study design

**Fig. 2:**
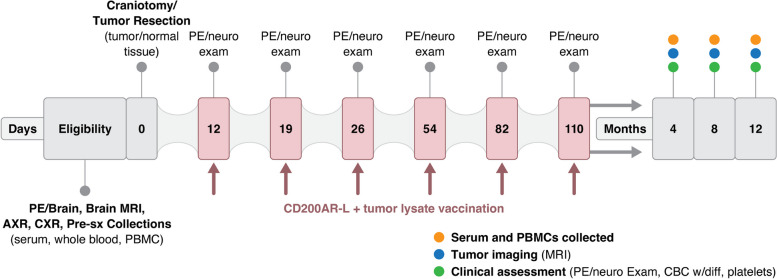
Comparative oncology trial schema for assessment of Cd200AR-L in dogs with spontaneous glioma. Confirmation of eligibility and baseline sample collections inclusive of brain imaging and immunologic correlates in peripheral blood were followed by craniotomy surgery for tumor resection and tissue collection. CD200AR-L combined with a tumor tissue lysate were administered via intradermal injection starting on post-operative Day 12 and continuing through Day 110. Dogs were monitored with serial imaging and clinical assessments along with immune monitoring until study exit. PE = physical examination, AXR = 2-view abdominal radiography, CXR = 3-view thoracic radiography. PBMCs = peripheral blood mononuclear cells, MRI = magnetic resonance imaging. Sx = surgery

The study schema is given in Fig. 2. Dogs underwent maximal safe resection of primary malignant glioma that was confirmed with histology and immunohistochemical staining. Ten to fourteen days after the craniotomy, canine-specific CD200AR-L (5 μg/kg) was injected intradermally on the nape of the neck on Day 1 and again on Day 2 in combination with autologous tumor lysate (~ 500 g of protein) after topical application of imiquimod (1 packet 5% cream/12.5 g). Injections were repeated weekly for 3 weeks, then every 4 weeks for 3 months, and then every 6–8 weeks for one year, tumor recurrence, or patient death. Blood was collected for serum, plasma and isolation of PBMCs prior to the craniotomy (pretreatment assessment) and at 4-month clinical rechecks that included brain MRI that was compared to the immediate postoperative MRI to assess tumor burden.

#### Deliverables

A total of 35 pet dogs with a definitive diagnosis of grade III or IV glioma were enrolled over a two-year period. The CD200AR-L peptide injections were well-tolerated, and no adverse events were noted. Addition of CD200AR-L to the standard autologous tumor lysate vaccine protocol increased the median overall survival time to 12.9 months compared to 6.8 months after treatment with autologous tumor lysate vaccines alone. The two-year progression-free survival rate was 30% in dogs that received CD200AR-L with the lysate vaccinations. In addition, radiologic evidence of tumor regression was noted in dogs with residual tumor after suboptimal resection. Serum levels of soluble CD200 may also be a biomarker that predicts tumor regrowth prior to the radiologic appearance of tumor.

Human use: how was it informed by canine comparative oncology data?

The lack of apparent toxicities and efficacy demonstrated by a highly significant increase in tumor control and survival time confirmed clinical responses in pet dogs with naturally occurring malignant glioma garnered enthusiasm for initiation of a Phase I dose-escalation clinical trial for recurrent glioblastoma in adults (NCT04642937).

#### Current drug status

In the human Phase I trial, at dose level 1 (*n* = 6), three patients had a favorable radiographic response of a partial response (*n* = 1) or stable disease (*n* = 2) within the first 3 months of treatment. Serum analysis showed an increase in CD8 + , CD4 + , natural killer (NK), and NK-T cells as well as increases in IgM and IgG3 levels. Preliminary RNAseq studies revealed an increase of M0 macrophage and caspase activity in post-treatment biopsy brain tissue. After a pause in recruitment, the trial is set to continue with dose escalation. OX2 Therapeutics has continued the clinical development of this immune checkpoint inhibitor.

Vignettes 3 and 4 describe the use of comparative oncology to advance translation of two oncolytic virus therapies. The first is an oncolytic Herpesvirus given by intratumoral (IT) administration), and the second is an oncolytic Vesicular stomatitis virus given for the treatment of disseminated or metastatic disease.

Oncolytic viruses (OVs) are a form of cancer immunotherapy designed to selectively replicate within and kill tumor cells and induce an inflammatory response resulting in the recruitment of immune cells to the tumor microenvironment (TME). This combination of tumor cell lysis with immune recruitment promotes presentation of tumor associated antigens to T-cells to generate antitumor immune responses [48, 49]. Several oncolytic viruses are being evaluated clinically for the treatment of patients with advanced malignancies, many in combination with immune checkpoint blockade (ICB) [50, 51].

### Vignette #3: Oncolytic HSV M032 virotherapy

#### Preclinical Summary

Glioblastoma multiforme (GBM) is the most common primary brain tumor in humans and carries a grave prognosis. Despite advances in surgery, radiotherapy, chemotherapy, and immunotherapy, average survival has increased no more than one month for every decade of research. Following surgical resection, greater than 10^9^tumor cells may remain in situ [52]. Recurrence is common, and median survival remains less than two years. Certain breeds of pet dogs spontaneously and sporadically develop high-grade gliomas that follow similar incidence, treatment, and outcome patterns as their human glioma counterparts. Conventional therapies for canine gliomas also have limited efficacy, further supporting the need for combination therapies tailored to the unique characteristics of these tumors and their immune-evading mechanisms.

Type 1 herpes simplex virus (HSV-1) vectors are innately neurotropic and coding region alterations have resulted in the development of a viral vector that is avirulent in post-mitotic brain tissue, cannot overcome innate anti-viral response of normal cells, and can only replicate in tumor cells. HSV M032 is a genetically modified oncolytic type 1 herpes simplex virus that expresses human IL-12, a T-cell activating cytokine, eliciting a potent immune-related inflammatory response [52, 53]. The IL-12 construct is inserted into two loci that previously had the diploid ϒ_1_34.5 gene (“neurovirulence gene”). Importantly, human IL-12 has been shown to be safe and effective in eliciting an immune response in animals, including dogs [54].

Produced under current good manufacturing practices (GMP), in compliance with FDA recommendations, and qualified by the NCI’s Novel and Experimental Therapeutics (NExT) Program for clinical use, M032 is cytotoxic to a panel of human glioma cell lines, but non-neurovirulent in immunocompetent or immunocompromised mice when delivered intra-cranially (equivalent to 4 × 10 [11] PFU in humans). M032 produces physiologic levels of biologically active human IL-12, as measured by ELISA and functional assays [55]. Toxicology and biodistribution in 36 primates (*Aotus nancymaae)*demonstrated safety and confirmed the increased ability of M032 to produce a modest (IL-12-induced) ongoing inflammatory response following intracerebral injection when compared to G207 (a non-IL12-producing HSV) studied in a similar model [55]. This species was chosen as they are highly sensitive to HSV, often terminally so. Two doses of HSV were tested – a low dose of 1 × 10^6^ plaque-forming units (PFU) and a high dose of 1 × 10^8^ PFU. On a dose/gram of brain basis, these doses would correspond to human equivalent doses of 5 × 10^7^ PFU and 5 × 10^9^PFU, respectively. Two male and two female NHPs from each dosing group were humanely euthanized at 3, 31, and 91 days. The animals underwent comprehensive necropsies as well as HSV PCR analyses to assess for the presence and copy numbers of the virus in various neural and systemic tissues. No serious toxicities or adverse events were identified that could be directly ascribed to M032. With respect to safety, M032 retains its sensitivity to acyclovir, an FDA approved anti-viral agent, which can be used to interrupt viral replication in the case of toxicity concerns [55]. Immunohistochemical data have demonstrated augmented inflammatory responses in the immunocompetent syngeneic models exposed to IL-12-expressing oHSV, suggesting that induction of a systemic immune response may at least partially account for anti-tumor responses [56].

In summary, the M032 oHSV utilized in the CANINE Trial (CANine ImmunoNEurotherapeutics) described below is the product of rigorous molecular engineering with stringent preclinical safety testing for intracerebral inoculation in immunocompetent or immunocompromised mice and non-human primates.

### Comparative Oncology Trial

#### Questions

Is intratumoral M032 administration—alone and in combination with an oral checkpoint inhibitor—safe, tolerable and effective in treating pet dogs with sporadic glioma? What systemic immune response is stimulated in dogs treated with an optimal dose of M032 and increasing doses of IDO inhibitor and can this be correlated with survival? Will sequencing of the intracranial gliomas identify potential target genes and predict response or toxicities to this immuno-oncoviral therapy?

#### Study design

**Fig. 3:**
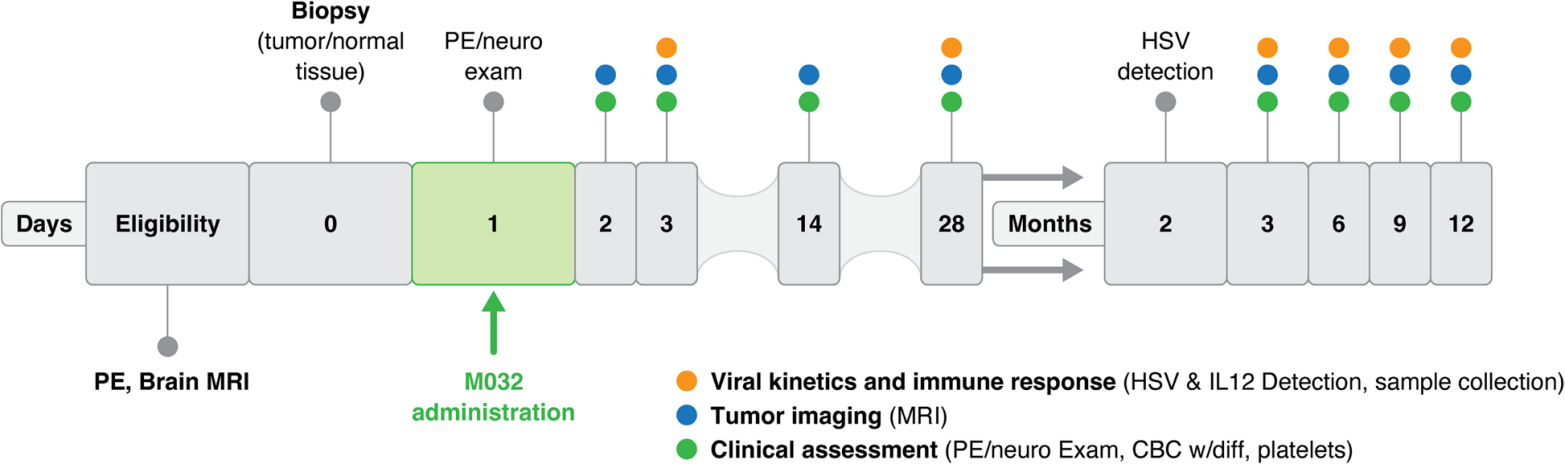
Comparative oncology assessment of oHSV-M032 in dogs with spontaneous glioma. In this study dogs underwent baseline assessment inclusive of tumor imaging, clinical laboratory testing, immunologic assays such as serum cytokines, HSV antibody titer and HSV detection in saliva and peripheral blood. Surgical placement of the intracranial catheter with CT guidance is performed along with tumor biopsy, followed by a single dose of oHSV-M032. Monitoring of clinical and immunologic parameters follows at regular intervals until study exit. PE = physical examination, MRI = magnetic resonance imaging, CBC = complete blood count, HSV = Herpes simplex virus

The study schema are given in Fig. 3. The CANINE Trial employs HSV M032 to treat canine glioma [57]. M032 has been proven safe for intracerebral administration in HSV-hypersensitive non-human primates at a per kilogram dose that far exceeds the dose employed in this study. First to employ oncolytic viral therapy for naturally occuring canine brain tumors and rooted in high quality pre-clinical data, this trial has established the safety and tolerability of oncolytic virus administration in the dog.

In Stage 1, now completed, pet dogs presenting with spontaneous gliomas underwent maximal safe tumor resection and inoculation of the tumor cavity with viral infusate. Stage 2 is ongoing and includes treatment in Stage 1 followed by oral Indoximod acting as a checkpoint inhibitor by blocking the immunoregulatory enzyme indoleamine 2,3 dioxygenase (IDO). IDO is expressed by high grade gliomas and essential for conversion of tryptophan to kynurenin, supporting immune evasion by the tumors.

#### Deliverables

In Stage 1 of the CANINE Trial, twenty-one canine glioma patients were enrolled between January 2018 and August 2020, each followed for up to one year. The majority of gliomas exhibited morphologic and immunohistochemical features consistent with oligodendroglioma (62%) and the remainder were identified as astrocytomas. Treatment-naive canine glioma microenvironment had enrichment of Iba1 positive macrophages and minimal numbers of T and B cells, consistent with previous studies identifying these tumors as immunologically “cold”.

NanoString mRNA profiling revealed enrichment for tumor intrinsic pathways consistent with suppression of tumor-specific immunity and support of tumor progression. Post-treatment changes included mRNA signatures corresponding with interferon signaling, lymphoid and myeloid cell activation, recruitment, and T and B cell immunity.

Multiplexed protein analysis identified a subset of oligodendroglioma subjects with increased concentrations of IL-2, IL-7, IL-6, IL-10, IL-15, TNFα, GM-CSF between 14 and 28 days after treatment, with evidence of CD4 + T cell activation and modulation of IL-4 and IFNγ production in CD4 + and CD8 + T cells isolated from peripheral blood.

To date, median overall survival from the date of treatment was 151 days (± 78 days) and no significant adverse events attributable to M032 or dose-limiting toxicities were observed.

Treatment with M032 did not cause harm and the combination of surgery and oncolytic viral therapy may have contributed to prolonged survival in pet dogs with spontaneous gliomas. Intratumoral treatment modulated immune responses, inducing changes in the tumor microenvironment (TME) and periphery consistent with priming innate and adaptive immune responses [58]. Results from dogs receiving Indoximod in Stage 2 and genomic sequencing of pre- and post-treatment tumors will be analyzed and translated for future human trials.

Human use: how was it informed by canine comparative oncology data?

Efficacy in the treatment of canine glioma confirmed clinical responses in naturally occurring disease and has provided pilot data and novel catheter methods with Rickham reservoirs—now in use in human high-grade glioma trials. In the CANINE Trial, viral administration was performed postoperatively via catheter to establish a more controlled volume, speed, and accuracy of delivery, with the patient awake to monitor for adverse reactions. The fully implanted catheter and Rickham reservoir were employed to allow for future re-dosing, serial sampling and to serve as a fiducial marker on subsequent imaging. Implantation also reduced the risk of accidental displacement.

#### Current Drug Status

M032 and related C134 HSV [59] re now being tested in humans with high-grade malignant gliomas. Eighteen patients have received treatment through two trials: M032 combined with Pembrolizumab to treat newly diagnosed or recurrent glioblastoma, anaplastic astrocytoma, or gliosarcoma in adults (NCT 05084430) and C134 HSV to treat recurrent GBM in adults (NCT03657576).

### Vignette #4: Oncolytic virotherapy using VSV-IFNβ-NIS

#### Preclinical Summary

Oncolytic Vesicular stomatitis virus (VSV), VSV-IFNβ-NIS, is an example of a clinical-stage OV therapy where clinical development was guided by comparative oncology studies in canine cancer. VSV is a negative strand RNA virus of the *Rhabdoviridae*family that naturally infects livestock including horses and cattle. Natural infections in livestock can cause vesicular lesions and are generally self-limiting. VSV is generally nonpathogenic in humans and natural infections acquired through contact with livestock or laboratory settings have been reported to cause mild, self-limiting symptoms [60]. VSV has a broad tropism and is able to infect most mammalian cell lines, including human and canine cells, with enhanced replicative capacity in cancer cells [61]. The absence of pre-existing immunity against VSV among the general human population supports the development of VSV as a systemic therapy that can be used to treat advanced and metastatic cancer. As modifications to optimize for systemic therapy, VSV was engineered to encode interferon-beta (IFNβ) to enhance tumor specificity by activating innate immune responses in normal cells, and the sodium iodide symporter (NIS) to allow noninvasive imaging of virus biodistribution [62]. VSV-IFNβ-NIS was tested in preclinical murine myeloma tumor models demonstrating that IV administered VSV is safe and results in durable tumor remission [63]. Serial noninvasive SPECT/CT imaging with a NIS specific radiotracer showed tumor specific amplification of VSV. Immunofluorescence analysis of explanted tumors provided a detailed view of VSV extravasation and radial expansion of intratumoral foci of VSV infection resulting in tumor killing [64, 65]. T-cell depletion resulted in mice with mostly incomplete tumor remission and relapse, indicating that complete tumor remission was immune-dependent. Studies subsequently showed that systemic VSV induces increased intratumoral CD8 T-cells resulting in improved therapeutic response to immune checkpoint blockade [66]. These promising preclinical results supported clinical development of oncolytic VSV, with the caveat these observations were in a VSV-susceptible murine model that provided an “ideal” scenario to model parameters that impact tumor elimination [64]. Predictably, preclinical efficacy varied in different murine tumor models [65–67], highlighting the need for studies in naturally occurring and heterogeneous tumors that better recapitulate human malignancies.

Prior to initiating a veterinary clinical trial in pet dogs with cancer, a dose-escalation study was carried out in healthy research beagles. Dogs were monitored closely for acute adverse events including monitoring vitals and clinical lab testing (blood chemistry, complete blood count (CBC), coagulation). Results showed that doses up to ~ 10^9^ TCID_50_/kg were well-tolerated and dose-limiting toxicities (DLTs) included acute and transient hepatotoxicity and lymphopenia [68]. Blood samples were also collected to monitor acute virus pharmacokinetics (PK) at baseline, 30, 60, 90-, 120-, 240-, and 360-min post infusion. Viremia and antiviral antibody response was monitored at 1-, 3-, 5-, 7-, 14- and 21-days following infusion. Virus PK and viremia was measured by qRT-PCR to detect VSV-N gene copy number. Peak viremia was dose dependent, with detectable VSV RNA staying fairly constant up to 24 h post infusion, and then rapidly declining to undetectable levels 7 days post infusion with a corresponding increase in anti-VSV neutralizing antibodies detectable in serum. Virus shedding was assessed in buccal swabs and excreta demonstrating the absence of infectious virus in urine, buccal swab, and fecal samples and supporting further studies testing oncolytic VSV in pet dogs with naturally occurring cancer.

### Comparative oncology trial

#### Questions

What are clinical toxicities associated with systemic VSV-IFNβ-NIS infusion in tumor bearing dogs? What is a safe starting dose range for VSV-IFNβ-NIS in humans with advanced malignancies? Is infectious virus shed following systemic administration in tumor bearing dogs? Does systemic VSV-IFNβ-NIS therapy induce tumor remission or provide clinical benefit in dogs with advanced malignancies? What cancer types are more responsive to systemic VSV-IFNβ-NIS therapy? Do virus pharmacokinetic measures correlate with clinical response (and serve as potential biomarkers of response)?

#### Study design

**Fig. 4:**
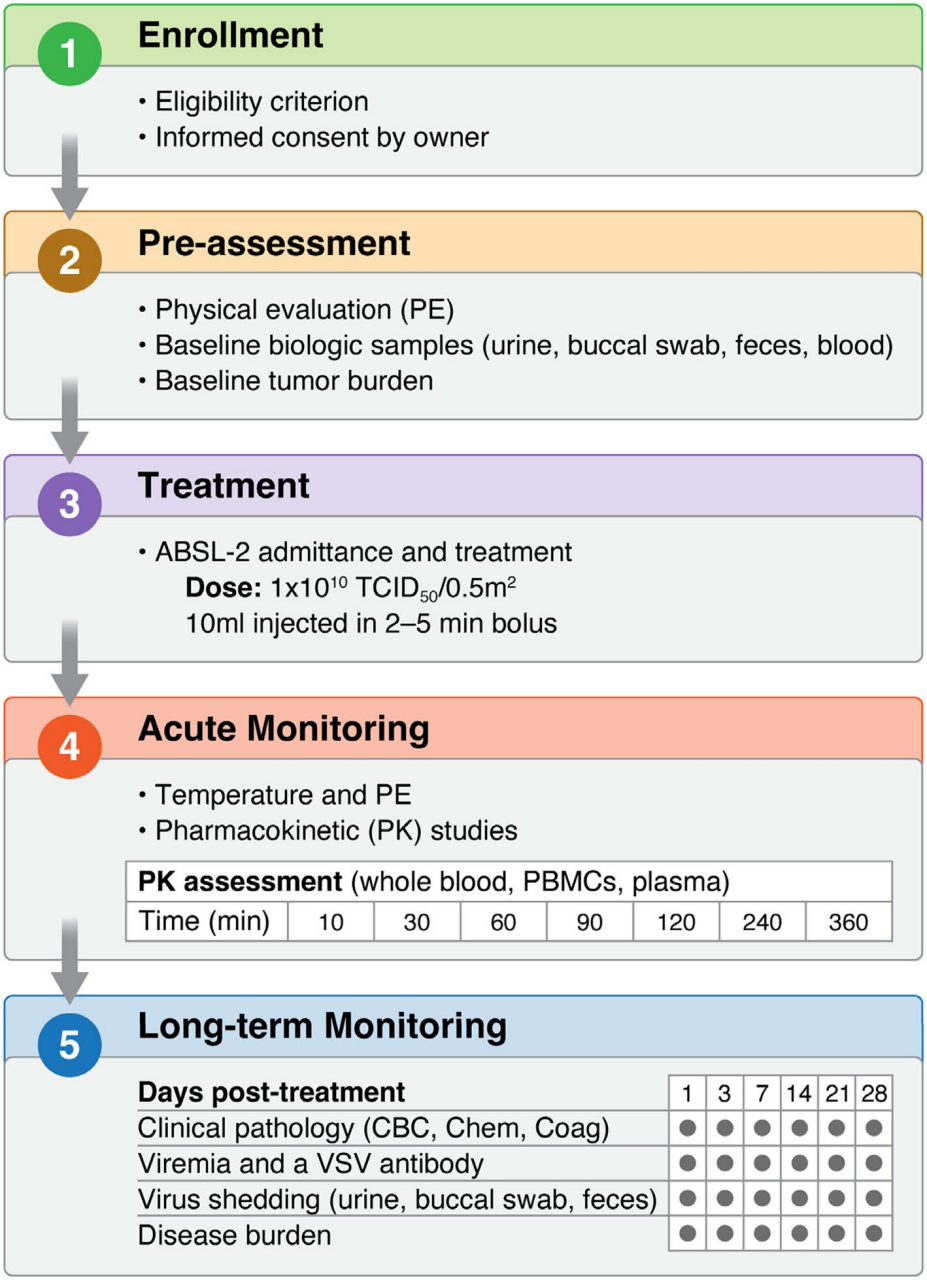
Comparative oncology assessment of VSV-IFNb-NIS in canine cancer patients. Following confirmation of eligibility, baseline assessment and collection of biologic specimens, a single intravenous dose of VSV-IFNb-NIS is administered within an ABSL-2 animal housing unit. Both acute and long-term monitoring are carried out to assess clinical and laboratory changes in response to virus administration. Disease burden and immune response data are collected over a 4 week period and beyond to determine response to therapy

Study schema are given in Fig. 4. For the first-in-dog veterinary clinical trial, a VSV-IFNβ-NIS vector expressing canine IFNβ was generated enabling comparison of the safety and efficacy of VSV expressing human versus canine IFNβ in dogs. In an all-comer study, a total of 9 dogs with advanced cancer were screened, enrolled, and received a single IV dose of 10^10^ TCID_50_/0.5m^2^ (~ 10^9^ TCID_50_/kg) VSV-IFNβ-NIS (expressing either canine or human IFNβ) [69]. Safety was assessed using Veterinary Cooperative Oncology Group-Common Terminology Criteria for Adverse Events (VCOG-CTCAE v2) [70]. Correlative studies included monitoring viremia, virus shedding, virus replication by measuring serum IFNβ, and antiviral antibodies. Virus PK and viremia in whole blood as well as blood separated into peripheral blood mononuclear cells (PBMCs) and plasma indicating that IV administered VSV localized to PBMCs (with VSV RNA below limit of detection in plasma). Infectious virus was detectable in isolated PBMCs up to 4 h post VSV infusion. Preliminary efficacy was assessed by measuring change in tumor burden during a 28-day period following VSV therapy with results showing partial response (PR) in *N* = 2 dogs (22%), stable disease (SD) in *N* = 4 dogs (44%), and progressive disease (PD) in *N* = 3 dogs (33%). Notably both responding dogs were boxers diagnosed with advanced peripheral T-cell lymphoma where single dose VSV infusion led to remission of all measurable lymph node lesions albeit transiently before disease relapsed. While IV VSV therapy was generally well-tolerated, the two responding dogs were outliers in terms of adverse events, developing grade 2 and 3 hepatotoxicity that resolved spontaneously. The two responding dogs also had the highest peak viremia and prolonged persistence of detectable human IFNβ in serum. These results collectively indicated that (i) T-cell lymphoma may be a promising target for VSV therapy, and (ii) that clinical response to systemic VSV therapy may be associated with hepatotoxicity and PK biomarkers including peak viremia and persistence of serum IFNβ. One dog with metastatic osteosarcoma (with pulmonary lesions) was enrolled and received two doses of IV VSV therapy given 48 h apart demonstrating that repeated VSV dosing was well-tolerated. This dog had stable disease for 6 months, a clinical outcome that was notable given that most dogs succumb to metastatic osteosarcoma 1–2 months following diagnosis.

#### Deliverables

This translational pathway demonstrated the utility of studies in naturally occurring canine cancer to guide clinical development of novel OV therapies assuming the requirements of tropism (i.e. that the OV agent can infect canine cells) and safety (i.e. the viral vector is safe in dogs) are met. The specific deliverables described include identifying target indications and reducing dose escalation steps, reducing the number of patients that receive subtherapeutic doses of investigational agent during dose escalation and increasing the likelihood patients gaining clinical benefit in the clinical trial setting. Additional studies were recently completed demonstrating the safety of oncolytic VSV administered in the neoadjuvant setting in canine osteosarcoma [71]. While the study cohort size precluded formal assessment of clinical efficacy, VSV-treated dogs had a high proportion of long-term survivors, an outcome associated with a T-cell anchored gene expression signature in resected osteosarcoma specimens. This study demonstrates the potential utility of comparative studies to identify genomic subtypes of cancer associated with clinical response and provides opportunities for future assessment of novel combination therapy approaches.

#### Human use: how was it informed by canine comparative oncology data?

The results from comparative oncology studies testing VSV therapy in dogs with naturally occurring cancer became part of the IND safety packet that was successfully approved by the FDA leading to launch of a first-in-human (FIH) VSV-IFNβ-NIS dose escalation study that followed a standard 3 + 3 design. A previous Phase I study testing IV oncolytic measles virus therapy in patients with advanced myeloma was initiated at starting dose of 10^6^ TCID_50_ [72]. In comparison, the VSV FIH trial was approved at a starting dose of 5 × 10^9^ TCID50 based on the demonstrated safety in dogs with advanced malignancies. This meant that fewer patients receive suboptimal VSV doses, and potentially therapeutic doses was more rapidly reached. The clinical responses observed in canine T-cell lymphoma prompted inclusion of T-cell lymphoma as an indication in the FIH studies. Fifteen patients were enrolled to the dose escalation phase of this study with no DLTs observed and a comparable adverse event profile to those observed in dogs. Dose level 4 (DL4), 1.7 × 10^11^ TCID_50_, was found to be the MTD [73]. Six patients were enrolled at DL4 with 5 of 6 patients experience SD (*N* = 3), PR or CR (*N* = 1 each). Notably 7 patients with T-cell lymphoma were enrolled at the 4 dose levels, with 3 of 6 patients showing a clinical response to VSV infusion (2 PR, 1CR) and SD in 3 patients. The clinical responses observed in patients with advanced T-cell lymphoma are notable given that T-cell lymphoma is generally considered a intractable malignancy. This study highlights the utility of comparative oncology studies to guide selection of target indications for clinical development.

#### Current Drug Status

Based on the promising phase I data, VSV-IFNβ-NIS is currently being tested in an expanded cohort of patients with T-cell lymphoma, and in a Phase I/II and II studies in patients with advanced solid tumors (NCT03647163 and NCT06508463) [74–76].

## Conclusions

Immunotherapy continues to gain traction as a key component in therapeutic strategies across many types of cancer. A comparative approach leveraging the canine cancer patient as a model species has demonstrable advantages and significant potential to inform this field. Future studies employing a comparative oncology approach are ongoing in many aspects of immunotherapy, including adoptive and genetically-engineering cell based therapies, immune synapse modulators, and combination approaches.

As with small molecule trials, inherent flexibility in comparative oncology trial designs supports expeditious investigations into altered dosing schedules that are informed by interim analysis of clinical and biologic data.

## Data Availability

No datasets were generated or analysed during the current study.
